# Detecting Cotton Leaf Curl Virus Resistance Quantitative Trait Loci in *Gossypium hirsutum* and iCottonQTL a New R/Shiny App to Streamline Genetic Mapping

**DOI:** 10.3390/plants12051153

**Published:** 2023-03-03

**Authors:** Ashley N. Schoonmaker, Amanda M. Hulse-Kemp, Ramey C. Youngblood, Zainab Rahmat, Muhammad Atif Iqbal, Mehboob-ur Rahman, Kelli J. Kochan, Brian E. Scheffler, Jodi A. Scheffler

**Affiliations:** 1 Bioinformatics Graduate Program, North Carolina State University, Raleigh, NC 27695, USA; 2 Department of Crop and Soil Sciences, North Carolina State University, Raleigh, NC 27695, USA; 3 USDA Agricultural Research Service, Genomics and Bioinformatics Research Unit, Raleigh, NC 27695, USA; 4 Institute for Genomics, Biocomputing and Biotechnology, Mississippi State University, Starkville, MS 39762, USA; 5 Plant Genomics and Molecular Breeding Laboratory, National Institute for Biotechnology and Genetic Engineering College, Pakistan Institute of Engineering and Applied Sciences, (NIBGE-C, PIEAS), Faisalabad 38000, Punjab, Pakistan; 6 School of Life and Environmental Sciences, The University of Sydney, Sydney, NSW 2006, Australia; 7 Institute for Genome Sciences and Society, Texas A&M University, College Station, TX 77843, USA; 8 USDA Agricultural Research Service, Genomics and Bioinformatics Research Unit, Stoneville, MS 38776, USA; 9 USDA Agricultural Research Service, Crop Genetics Research Unit, Stoneville, MS 38776, USA

**Keywords:** genetic mapping, cotton, genotyping, R/Shiny, cotton leaf curl virus, quantitative trait loci

## Abstract

Cotton leaf curl virus (CLCuV) causes devastating losses to fiber production in Central Asia. Viral spread across Asia in the last decade is causing concern that the virus will spread further before resistant varieties can be bred. Current development depends on screening each generation under disease pressure in a country where the disease is endemic. We utilized quantitative trait loci (QTL) mapping in four crosses with different sources of resistance to identify single nucleotide polymorphism (SNP) markers associated with the resistance trait to allow development of varieties without the need for field screening every generation. To assist in the analysis of multiple populations, a new publicly available R/Shiny App was developed to streamline genetic mapping using SNP arrays and to also provide an easy method to convert and deposit genetic data into the CottonGen database. Results identified several QTL from each cross, indicating possible multiple modes of resistance. Multiple sources of resistance would provide several genetic routes to combat the virus as it evolves over time. Kompetitive allele specific PCR (KASP) markers were developed and validated for a subset of QTL, which can be used in further development of CLCuV-resistant cotton lines.

## 1. Introduction

Cotton is an important crop grown around the world for use in textiles, livestock feed, and human consumption [1]. Cultivated cotton, *Gossypium hirsutum*, is an allotetraploid crop that accounts for much of the fiber used in natural textiles. In addition to its uses in clothing and other textile products, cotton is used in the production of American currency [2]. Due to its high yield and versatile uses, cotton is a major export and source of income for farmers in many countries.

Pakistan is the 4th largest producer of cotton in the world, where cotton-textiles account for 11 percent of the gross domestic product and over 60 percent of export receipts [3]. Pakistan has recently been affected by the re-emergence of the cotton leaf curl virus (CLCuV), which is the causative agent for cotton leaf curl disease (CLCuD). CLCuV was identified in the 1960s as a possible threat to cotton crops [4,5], and first arrived in agricultural areas of Multan, Pakistan in the 1980s, seriously affecting yield [6]. The Multan strain of the virus was mitigated with resistant varieties until the early 2000s when the new Burewala strain overcame the resistance previously bred into cotton varieties [4]. This virus is composed of a single-stranded DNA begomovirus (family Geminiviridae) containing a circular DNA A component and two satellites, which are designated as the alphasatellite and the betasatellite (DNA β). DNA β has been determined to be responsible for causing disease symptoms [6,7,8].

The virus causes severe economic loss to countries in affected regions. For example, the effects of this disease on production in Pakistan typically show a decrease of 2 to 3 million bales of lint, resulting in billions of dollars in loss for the country yearly [4,9]. In the last ten years, the disease has also been reported in neighboring countries, i.e., India and China, and is a potential threat in all cotton growing countries where its vector, *Bemisia tabaci*, the silverleaf whitefly, is prevalent [10]. The virus and its whitefly vector have many crop and ornamental hosts in addition to cotton, and there is concern that the virus, via its vector, will move into currently unaffected countries before resistance can be bred into cotton lines [1,8]. Plants infected with the virus early in the growing season exhibit stunted growth, leaf curling, and inhibition of flower development and subsequent cotton fiber production [7].

Development of lines through breeding resistance to CLCuV depends on screening each generation of plants in Pakistan where the disease is endemic. This greatly increases the amount of time needed to breed resistant lines, particularly due to legal difficulties with moving plant materials across borders. Identification of DNA markers linked to CLCuV resistance would allow breeders in other countries to develop resistant lines without the need for early disease screening. This proactive breeding of resistant lines in currently unaffected countries is essential to prepare for potential spread of the virus.

Previous efforts to develop markers have not been successful due to limited genetic diversity among cotton lines and a lack of usable DNA-based markers. As reviewed previously [8], researchers attempted to identify restriction fragment length polymorphism (RFLP) markers associated with the Multan strain of CLCuV and reported three potential RFLP markers, but they did not transfer to other populations. In 2002 and 2005, researchers explored using random amplified polymorphic DNA (RAPD) markers, and three were identified associated with the source of resistance to the Multan strain, but they did not prove to be consistent. Efforts to identify and verify simple sequence repeat (SSR) markers associated with the emerging Burewala strain of CLCuV have been ongoing since 2012, and this study was initiated in 2013 to find SSR or single nucleotide polymorphism (SNP) markers associated with the new strain of CLCuV. Starting in 2015, SNPs have become more available and there have been a number of high-density genotyping platforms developed for cotton, including the CottonSNP63K with over thirty-eight thousand Functional Polymorphic markers available [11,12].

While there are limited published reports of usable DNA markers associated with CLCuV, there are some studies that provide insight into the possible mechanism of resistance, chromosomal location of resistance, or the genes responsible. Diploid relatives of tetraploid cotton are resistant to both the Multan and Burewala strains of CLCuV. Two studies demonstrated through grafting experiments that diploid cotton could be infected, but the virus did not increase or spread significantly within the plant [13,14]. The Naqvi et al. study [14] used transcriptome analysis to identify genes in diploid *G. arboreum* that may be responsible for the resistance, and a study by Zaidi et al. [15] confirmed that many of the same genes were responsive in tetraploid cotton. While putative genes have been localized to all 26 chromosomes through transcriptome analysis, multiple published studies indicate that genetic resistance is simply inherited, indicating that only a few genes can condition high tolerance/resistance [16].

Recently Vij et al. made a cross between a synthetic tetraploid created from resistant diploids and a susceptible tetraploid *G. hirsutum* [16]. They phenotyped segregating progeny from the cross, and then used genotyping by sequencing to identify SNP markers associated with the resistance trait originating from the diploid source. They reported that there were two QTL responsible for the resistance found on A01 (chromosome 01 or 15) and D07 (chromosome 07 or 16) [16]. These QTL were associated with three SNPs that they plan to evaluate as putative markers for marker-assisted selection.

Wild tetraploid landraces and germplasm lines (*G. hirsutum*) have also been found to be a valuable source of resistance to many diseases because they tend to retain genes for resistance that may have been lost in the cultivated lines [8]. These landraces are often photoperiod-sensitive, which need short days to flower and set seed under day lengths found near the equator [8]. One germplasm line, Mac7-0238 [15], an adapted day-neutral line, has been identified as a potential source of resistance.

This study aims to determine markers closely linked to genes available in tetraploid *G. hirsutum* that control resistance to the cotton leaf curl virus. Identifying markers will allow breeders to use marker assisted selection (MAS) to more effectively select resistant lines, potentially without the need to screen the materials on location in Pakistan in early generations. This project was designed to determine if resistance is linked to the same genetic regions across different sources of resistance or if there are multiple genes that can be utilized in the germplasm. Specifically, this study investigates whether resistance in Mac7-0238 is caused by genes in the same genomic location as the resistance observed in a set of additionally identified resistant Marie Galante (moco) landrace lines. Given the virus’ history of evolving to overcome past successes at breeding resistance in the past, breeding in multiple different resistance genes would make it much harder for the virus to overcome resistance in the future.

## 2. Results

### 2.1. Phenotyping

**Figure 1:**
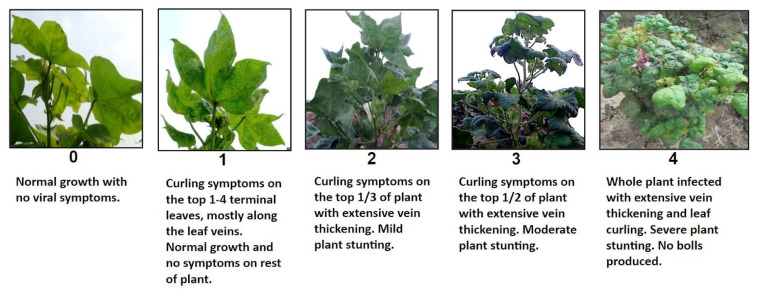
Disease scale used to score for cotton leaf curl virus (CLCuV) symptom severity. Plants were rated on a scale of 0 to 4, where 0 = no symptoms of CLCuV and 4 = severe leaf curling and very reduced boll set.

**Figure 2:**
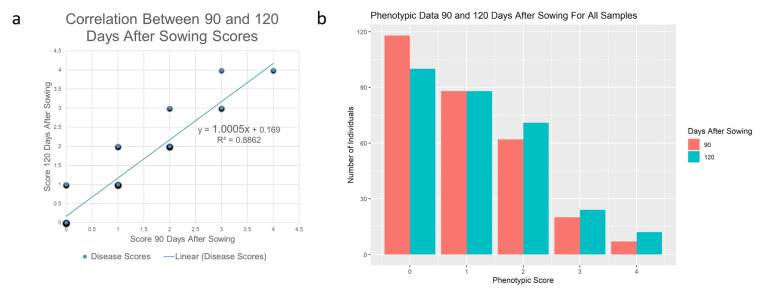
Summary of phenotyping for cotton leaf curl virus (CLCuV) of mapping populations. (**a**). Correlation graph between the disease ratings for CLCuV at the two time points of observation. Rating scale of 0 to 4, where 0 = no symptoms and 4 = severe curling and very reduced boll set. Size of plot points indicates the number of individuals with that given rating. (**b**) Distribution of phenotypes across all the populations at 90 and 120 days after sowing.

Plants were rated for disease resistance at 90 days after sowing (DAS) and 120 DAS on a scale of 0 to 4, where 0 = no symptoms of CLCuV and 4 = severe leaf curling and very reduced boll set (Figure 1). Susceptible controls planted in an organized grid pattern throughout the field test were found to consistently behave as expected with severe ratings (i.e., 4). The correlation of ratings between the two time points of the segregating F_2_ plants were analyzed (Figure 2a). Given that the R value of the Spearman test was 0.94138 and the R^2^ value was 0.8862 when comparing the difference between the phenotypes at 90 days after sowing and 120 days after sowing, a positive linear association was observed between the two time points. If any ratings changed, they typically were increased by a single value in the later rating, as seen in Figure 2b and Supplementary Figure S1F or these reasons, the results from day 120 were used for QTL mapping to allow susceptible plants to have sufficient time to be infected and show symptoms.

### 2.2. Genetic Mapping

In order to identify genomic regions related to resistance to CLCuV, six F_2_ populations were created from four crosses between different sources of resistant material and susceptible genotypes. Resistant genotypes were selected from different sources: wild landraces, TX1214 and TX1145, and the adapted day neutral germplasm line Mac7-0238. Known susceptible lines, MD2ne and Mac7-1238, were used as the other parents of the crosses (Table 1). Plants of the populations and a select group of individuals of each parental line were genotyped on the CottonSNP63k array [11]. Markers that were polymorphic between the parents of the cross were used to create genetic linkage maps in Joinmap (Table 1). Small population sizes resulted in the estimated linkage sizes being much larger than expected, making it necessary to compare to previous literature to determine if the linkage map marker order was reasonable and followed the results of the larger studies (Supplementary Materials, Figure S2). Checking against previous literature ensured that the marker order was reasonable even if the lengths of the maps were expanded [11]. This process also allowed for determination of which chromosomes (using the 1–26 chromosome designation) were associated with the individual linkage groups. Filtered markers were used to run Single Marker Association testing with PLINK [17,18]. After using the Bonferroni correction method for multiple comparisons, none of the markers for any of the populations remained significant.

Filtered markers from Population 1 grouped into 70 linkage groups with a span of 20,389.14 cM and an average linkage group size of 784 cM. Populations 2 and 3 were grouped together based on the same parentage, creating a genetic map of a span of 29,700.5 cM across 33 linkage groups and an average size of 1188.02 cM. Initially, Populations 4 and 5 were combined for a total of 57 linkage groups; however, a majority of groups overlapped in physical space on the chromosomes when compared to previous literature instead of binning into the same group, and further analysis with the populations merged in this way did not produce significant results. Therefore, these populations were separated, which resulted in 97 linkage groups spanning 19,069.27 cM with a 733.43 cM average for Population 4 and 56 linkage groups spanning 57,905.5 cM with an average of 2227.13 cM for Population 5. While the number of groups is larger and linkage group size expanded, the split produced groups of markers where marker orders were more similar to previous literature without group overlap [11]. Population 6 had a total of 42 linkage groups spanning 19,513.4 cM with a 750.5 cM average. All populations were observed to have corresponding linkage groups for each of the 26 cotton chromosomes, except the grouped populations 2 and 3 where all but Chromosome 20 were represented (markers from this chromosome did not sort into a group of 10 or greater markers) (Supplementary Materials, Table S1).

**Figure 3:**
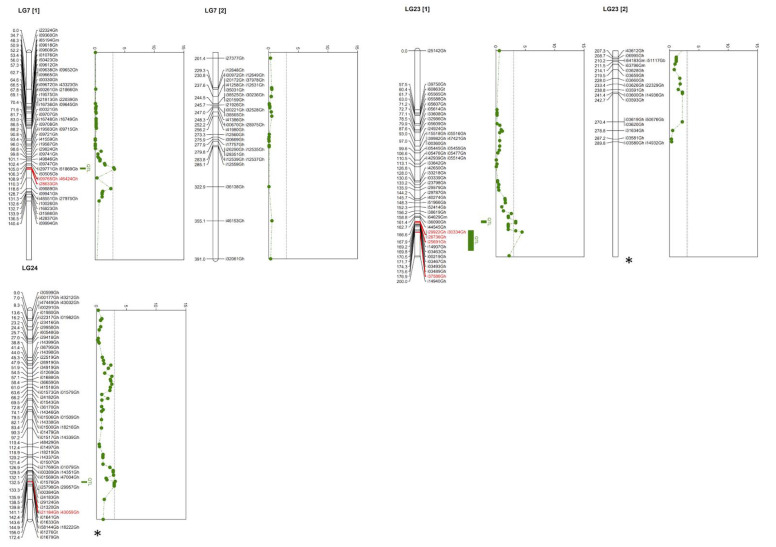
Quantitative Trait Loci identified for Population 6. Linkage Groups 7, 23, and 24 shown for Population 6. Markers associated with QTL are colored in red, and the QTL interval is shown as a green bar to the right of the linkage group. Graph of the likelihood of odds scores (LOD) for each marker is shown to the right of the corresponding linkage group. The dotted line on each graph shows the LOD threshold at 3.0. * Indicate a cut off portion of the linkage group map is not shown.

Using these genetic maps, several QTL were identified across the different populations (Table 2). It was notable that different QTL were identified in each of the populations. For the purposes of this project, an LOD score of 3.0 was considered significant. QTL were identified for each of the populations, totaling 29 markers. Population 1, MD26ne × TX1214, identified two markers from Chromosome 8. Combined Populations 2 and 3, Mac7-1238 × TX1145, identified 11 markers from Chromosomes 9, 10, 15, and 21. Population 5, MD26ne × TX1145, identified six markers from Chromosome 16. Population 6, MD26ne × Mac7-0238, identified 11 markers from Chromosomes 3, 5, and 16 (Figure 3). Detailed results are shown in Supplementary Materials, Table S2.

### 2.3. Marker Assay Development and Validation

A subset of QTL and surrounding markers were selected to test for conversion to Kompetitive allele specific PCR (KASP) marker assays. The subset consisted of all the QTL from Population 5 (MD26ne × TX1145 cross) and an additional four markers closely linked to the QTL and a subset of the QTL from Population 1 (MD26ne × TX1214 cross) as well as an additional closely linked marker to the two QTL selected (Table 3). Non-QTL markers were selected from a pool of closely linked markers to the QTL that were successful in the conversion to KASP markers. Overall, we tested eleven KASP marker assays on a new set of samples obtained in the US, and found eight to be of sufficient quality to use moving forward (Table 3). This represented a 72.72% success rate of conversion of assays to KASP, in line with the success rate of other marker conversions to KASP [19]. Of these, seven were found to behave in a co-dominant fashion and only one was found to behave in a dominant fashion.

**Figure 4:**
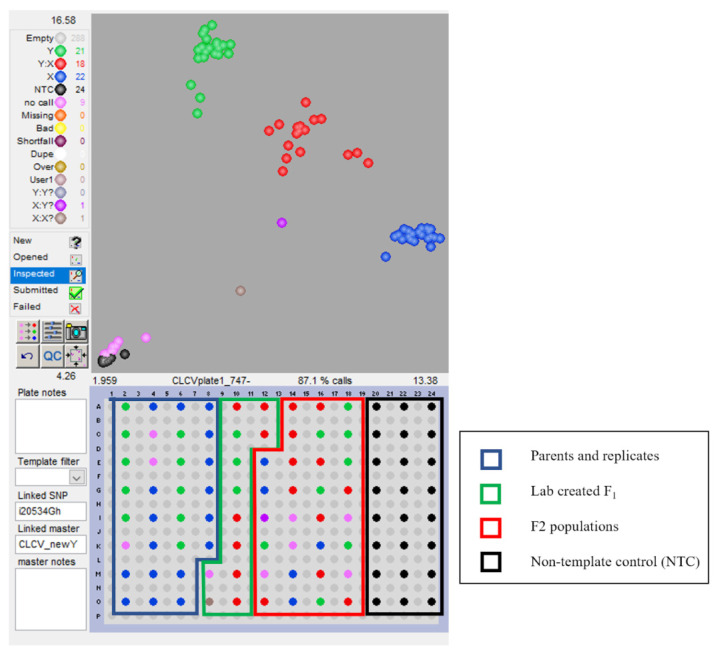
Overview of screening the KASP primer for marker i20534Gh. Image shown at PCR cycle 49 is an example of a selected “good” marker. The top half of the figure shows the clustering of homozygous individuals from the panel from the heterozygous individuals. The design of the testing panel is in the bottom half of the figure with boxes drawn to show sample type.

From the MD26ne × TX1145 cross, markers i01975Gh, i20534Gh, i38904Gh, i35622Gh, and i01767Gh were selected for reasonable clustering due to clear separation between X:X, and Y:Y clusters. Markers i20534Gh and i38904Gh separated well for X:X, X:Y, and Y:Y groupings with i20534Gh having tighter clusters (Figure 4 and Supplementary Materials, Figure S3). i01975Gh and i35622Gh did not segregate from the heterozygous cluster, the Y:Y cluster separated cleanly in i01975Gh, and the X:X in i35622Gh, of which both clusters are the resistance-containing individuals, making the marker still usable as a dominant marker for homozygous calling of resistant individuals. i01767Gh did not separate out as well between the resistant group (X:X homozygous in this plot) and the heterozygous individuals; however, this may cluster better with a larger population.

For the MD26ne × TX1214 cross, all the markers selected for the panel, i04503Gh, i60979Gt, and i64854Gm, showed reasonable clustering. The group of known resistant individuals in the testing panel for i60979Gt and i64854Gm both separated well away from the heterozygous and known susceptible individuals (X:X for i60979Gt and Y:Y for i64854Gm).

### 2.4. R/Shiny App iCottonQTL

**Figure 5:**
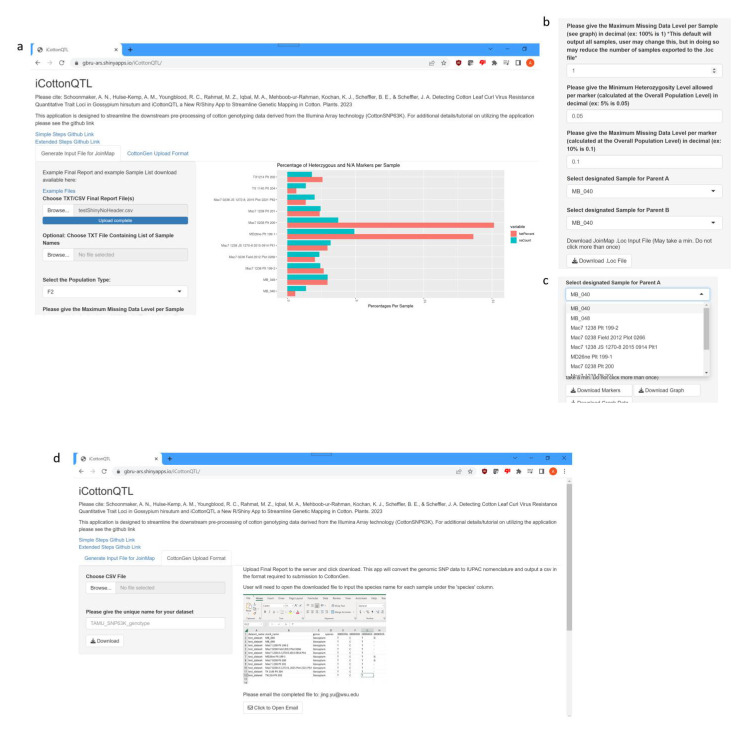
Screenshots of the iCottonQTL R/Shiny App. The application is available at https://gbru-ars.shinyapps.io/iCottonQTL/ (accessed on 21 February 2023). (**a**) Screenshot the first tab of the app, “Generate Input File for JoinMap” after loading test data. Depicts the graph of percentages of markers out of the set of Functional Polymorphic markers of each sample that are heterozygous or missing that loads upon upload. To the left is a panel where the user can edit parameters for filtering the markers. Parameters shown are an optional upload for a text file of a list of samples if the Final Report contains more than one project. (**b**) Screenshot of the additional parameters available for filtering samples and markers. Parameters include filtering samples for heterozygosity and missing data rates, with default percentages set in decimal form at 0.05 and 0.1, respectively. (**c**) Screenshot depicting the drop-down menu of samples to select parents of the cross. (**d**) Screenshot of the second tab, “CottonGen Upload Format”, which, after uploading the data from the CottonSNP63k array [11], outputs a comma-delimited file in the format required for data upload to the CottonGen website. Instructions for submission are included to the right.

Due to the repetitive nature in performing genetic mapping, we developed several R scripts to speed up the initial steps of the analysis. To better streamline analysis and assist the cotton research community, we bundled the R scripts into a publicly available Shiny App, iCottonQTL (Figure 5, https://gbru-ars.shinyapps.io/iCottonQTL/ (accessed on 21 February 2023)). This App allows a user to automatically process and convert the data from the Final Reports off the CottonSNP63K array [11] into the standard IUPAC format for submitting data to the CottonGen website, partition specific datasets from Final Report file(s), and convert filtered data from mapping parents and F_x_ samples of a cross into a JoinMap formatted .loc file for further analysis.

We developed iCottonQTL to be robust; in one tab, the user can take one or more Final Reports from the CottonSNP63k array along with an optional text file list of sample names for subsetting (should the project have been genotyped with other projects) for transposing the genotype data into the .loc ABH genetic file input for JoinMap and to allow the user to make choices on filtering the data. The App automatically filters down to the 38,822 Functional Polymorphic markers as identified in Hulse-Kemp et al. (2015). The user is then given the option to decide the levels of heterogeneity and missing data as cutoffs for each of the markers according to the population levels. The iCottonQTL interface provides a graph of the percentages of heterozygosity and missing data of the samples (Figure 5a). Defaults in decimal form are provided at 0.05 (5%) minimum heterozygosity and 0.1 (10%) maximum missing data for each of the markers at the population level, but can be changed by the user. The user must then select from a drop-down menu the samples corresponding with Parent A and Parent B (Figure 5b). Once these parameters have been selected, the user can download the .loc file containing the set of markers filtered according to the parameters and Fx genetic data converted according to whether the SNP matches Parent A, Parent B, or is heterozygous. Optional downloads include the ability to download the graph generated by the app (the graph displayed will limit the number of sample names visible for simplicity, but the graph downloaded will be large enough to display all the sample names), and the calculated data used to display the graph. Additionally, an option is included to download a comma-delimited file of the entire sample set filtered for the 38,822 Functional Polymorphic markers for manual manipulation or further custom analysis. In the other tab (Figure 5c), the user can upload a Final Report and download the file in the standard IUPAC form for upload to the CottonGen database (https://cottongen.org (accessed on 21 February 2023)). The app was tested with previously published data [20] to verify the results of the filtering step.

## 3. Discussion

### 3.1. Genetic Mapping Was Able to Identify Regions Associated with Disease Resistance

The use of high-throughput genotyping technologies is an important tool in determining genetic regions related to genes of interest. This study developed and experimented with methods in an attempt to deal with multiple difficult issues when trying to develop and evaluate the genetics associated with valuable plant materials generated with unadapted and/or photoperiod-sensitive parental lines. Developing populations with these types of materials is very difficult and traditionally produces low numbers of seed, particularly derived from a single F_1_ plant, as required for genetic mapping in a single population. Even when grown at locations suitable for flowering, it is difficult to get more than a few bolls off the F_1_ plants derived here due to their perennial nature. Thus, we utilized multiple small populations and investigated combining smaller populations.

In this project, CottonSNP63K was utilized to genotype six populations from four crosses. Populations were analyzed both as separate populations and as combined populations for each cross. It was determined that Populations 2 and 3 should be combined due to increased ability to identify linkage and Populations 4 and 5 should remain separate for the analysis, as the combined populations led to genetic maps that were prone to broken linkage when compared to positions of markers in previous literature. While both populations came from a cross between MD26ne and TX1145, combining Populations 4 and 5 together also did not identify any significant markers. Separating out the populations led to Population 5 identifying six significant markers on Chromosome 16, while Population 4 identified none. Closer examination of the phenotypic data for that population showed that while there were representatives of every part of the scale, except the most severe, at 90 DAS, all of the plants in the population showed signs of disease progression at 120 DAS (Supplementary Materials, Figure S1), suggesting that none of the plants were resistant to CLCuV, which explains the lack of QTL results when searching for a cause of resistance. Looking into the similarities between the individuals in Populations 4 and 5, a pca of the polymorphic markers between the two parents showed the individuals in Population 5 clustered together and apart from those of Population 4, unlike the pca of the combined Populations 2 and 3, which showed individuals from both populations clustering together in smaller groups (Supplementary Materials, Figure S4). Multiple reasons could exist for this. As it is not possible to identify visually the parents for a progeny plant, Population 4 may have been misidentified, and not have the same parents as Population 5 or be an outcrossing and only have the susceptible parent in common.

Each of the selected population combinations displayed good collinearity between the genetic maps for the population analysis and the agreed-upon positions from previous studies. The agreed collinearity shows the accuracy in linkage between markers, which allowed for trust in the maps even when the calculated distances between markers was higher than expected. We identified a region on chromosome 8 explaining up to 51% of the resistance trait in the MD26ne × TX1214 cross. Regions on five chromosomes account for between 17 and 30% of the trait in the cross between Mac7-1238 and TX1145. One chromosome explained up to 55% of the trait in the cross between MD26ne and TX1145 and the cross between MD26ne and Mac7-0238 identified regions on three chromosomes explaining up to 50% of the trait.

We created a KASP primer assay for a subset of the markers associated with the QTL as well as a few of the markers surrounding the QTL. Of these 11 markers we were able to select 8 markers for clear clustering on the test panel. Overall, parent samples in the testing panel segregated consistently with the expected allele for the genotype. These identified makers can be used for future screening of progeny for resistance without the need for testing each generation in locations where the disease is endemic and there is consistent disease pressure during the growing season. Future studies will include testing additional larger populations using this assay along with the phenotypic data from those populations.

### 3.2. Multiple Independent Sources of Resistance Indicated

Each of the populations identified different sources of resistance, hinting at the possibility of multiple resistance genes available in this cotton germplasm. The QTL identified from Populations 2 and 3 and Population 5, which share the same resistant parent, were not the same nor on the same chromosome. This is being investigated by making new F_2_ populations to confirm that this was a true difference and not a mislabeled population. Similarly, Populations 5 and 6 both identified regions on Chromosome 16, though not in the same region. The identification of genetic regions in the wild landraces, TX1145 and TX1214, that differ from those identified in the germplasm line Mac7-0238 indicates the potential of having multiple genes that can be combined and introduced into cotton varieties. If these QTL act independently from each other, the availability of multiple sources of resistance provides breeders the opportunity to stack multiple resistance genes in one variety.

Preliminary research (unpublished, Scheffler 2014 and 2017) with additional MD26ne × Mac7-0238 and MD51ne × Mac7-0238 F_2_ mapping populations screened with polymorphic SSR markers identified QTL on Chromosome 7 and/or 16 (BNL 1597) and one on Chromosome 3 and/or 14 (CIR 228). These data support the results found in the current study. It is likely that there are multiple genes required to have the highest level of resistance, but each one can improve tolerance to CLCuV. It appears the photoperiod lines TX1214 and TX1145 may have a different source of resistance compared to Mac7-0238. Given that resistance has been overcome in the past, this presence would provide a stronger defense against viral infection that may be more difficult to overcome in the future, especially if the resistance from each source has a different mode of action. The QTL and qualitative inheritance of the resistance source reported by Vij et al. [16] support our findings where QTL were also found on Chromosomes 15 and 16.

### 3.3. App for Streamlining Cotton Genetic Mapping

Genetic mapping in cotton has been greatly expedited recently with the availability of standardized genotyping platforms [11,21]. However, the process of utilizing the genotyping data to actually perform genetic mapping still requires a fair amount of data manipulation that can be a bottleneck to many. The ability to reformat scripts developed as a part of routine analyses into Graphics User Interface (GUI)-based applications such as R Shiny allow for movement of scripts from an individualized custom analysis to general tools, which can be utilized by research communities. As many thousands of samples have been and will continue to be run on array genotyping technologies, tools such as the iCottonQTL app will serve the cotton research community in the future. Hosting the scripts behind iCottonQTL on github (https://github.com/USDA-ARS-GBRU/Cotton_CottonLeafCurlVirus_QTLmapping (accessed on 21 February 2023)) will allow for future development and capacities as needed by the research community. While the app does not currently support the production of a large number of formatted outputs, the capacity was included to allow the user to collect sample data from a possible set of pooled projects, visualize the heterozygosity of the samples, and download the initial filtering set for Functional Polymorphic markers for further manipulation. These further manipulations can then be adapted to potentially be added to iCottonQTL in the future.

## 4. Materials and Methods

### 4.1. Plant Material and Phenotyping

Six F_2_ populations were created from bi-parental crosses using one CLCuV-susceptible and one resistant cotton line. The resistant parents came from either adapted germplasm Mac7-0238 (GVS9, ARS Release P.0063.14), or wild photoperiod-sensitive Marie Galante landraces TX1214 (PI 376039) and TX1145 (PI 284955) that originated from northeast Brazil. Population 6, created from a cross between MD26ne (PI 666042) and Mac7-0238, and Populations 2 and 3, created from a cross between MD26ne and Mac7-1238, shared one parent from the same lineage. Both Mac7-0238 and Mac7-1238 are selections from the original Mac7 (Gl 3 rai) [22], which was a line segregating for a number of morphological traits. When the two lines were evaluated in field screening tests for resistance to CLCuV, Mac7-0238 was scored as resistant and Mac7-1238 was scored as moderately susceptible. The initial crosses and the F_1_ generation were grown at the cotton winter nursery in Tecomán, Mexico (2013 and 2014), where the photoperiod-sensitive lines would flower normally. The F_2_ seed, produced at the cotton winter nursery, was sent to Pakistan and grown in a field screening nursery (2016) to evaluate the progeny for resistance to CLCuV. The F_2_ populations were planted about six weeks later than normal in mid-June (2016) so the plants would be younger and more susceptible to the virus. Plants were planted in a block with 1 m between rows and 45 cm between individual plants. Susceptible cotton lines were also planted in an organized grid pattern throughout the field test, as controls, to allow an assessment of the severity of the CLCuD infestation. The plants were rated at 90 and 120 days after sowing (DAS). Plants were rated on a scale of 0 to 4, where 0 = no symptoms of CLCuV and 4 = severe leaf curling and very reduced boll set (Figure 1). Before this rating scale was developed, each researcher had their own rating system with the number of classes ranging from 6 to 60. This standardized scale was developed as part of the collaborative Cotton Productivity Program to allow data collected by all collaborators to be easily combined and understood. The correlation of ratings between 90 and 120 DAS was evaluated to determine the best way to use the phenotypic data.

### 4.2. DNA Extraction and Genotyping

Young leaves were sampled from each of the individual F_2_ plants and multiple separate individuals for the parental lines (one to four individuals of each genotype were selected to represent the parents of each cross) [23]. Tissue was disrupted according to the protocol for TissueLyser II and DNA was extracted using a Qiagen Plant DNeasy kit following manufacturer’s protocol. Extracted DNA was assessed for quality using a Nanodrop Spectrophotometer and quantified using Picogreen. The parental plants and the F_2_ populations were genotyped on the CottonSNP63K array [11]. Genotype calls from the CottonSNP63K were produced using the standardized cluster file for the array as documented in the standardized operating procedure on CottonGen (https://www.cottongen.org/data/community_projects/tamu63k (accessed on 21 February 2023)).

### 4.3. Data Quality-Control and Filtering

A novel R script was developed to automatically convert Illumina’s default Final Report Files from CottonSNP63K array genotyping into a standard IUPAC nucleotide base format: https://www.cottongen.org/data/community_projects/tamu63k (accessed 21 February 2023). The script was developed to automatically produce a standard output required for uploading raw genotyping information to the CottonGen database. The data for this project were run through this pipeline and deposited into CottonGen.

Two additional R scripts were developed to automate CottonSNP63K array data for genetic mapping. The first script partitions the 38,822 Functional Polymorphic markers from the raw genotyping information and then the second converts those to the standard mapping file format for JoinMap (https://github.com/USDA-ARS-GBRU/Cotton_CottonLeafCurlVirus_QTLmapping (accessed on 21 February 2023)). Utilizing these scripts, heterogeneity was calculated for each of 1–4 individuals representing each of the mapping parental lines used in this study, out of the 38,822 Functional Polymorphic markers, to determine the level of homozygosity for each individual. Any plants assayed from the designated parent lines which had a similarity to other individuals of that parental line group of less than 80% were excluded from additional analysis. A consensus genotype for each parental line was then derived from the remaining individuals. These three R scripts were then combined into a Shiny App. One tab accepts Final Reports from the CottonSNP63K array and outputs .loc files for running analysis in JoinMap. The app acts as a general tool for projects genotyped on this array (although the code is general enough that other arrays can be added in later) to select samples from the project according to the user’s discretion, filter markers for functional polymorphism, missing data, and heterogeneity, and create next-step software files (again, capability is limited currently to JoinMap, but other options can be added later). The other tab converts data from the Final Report into the correct input for uploading to the CottonGen database in one step with a link to the CottonGen database for easy upload.

For each cross, the markers were first filtered for Functional Polymorphic markers. From this, markers were then selected if the markers were homozygous and the same across each set of individuals representing parental lines and were polymorphic between the two parental line sets. The heterozygosity across the population and the heterozygosity of each marker were calculated to check against the expected for F_2_ populations. The markers in the F_2_ populations were then translated into ABH format.

### 4.4. Map Construction

For analysis, the six separate populations of F_2_ created from the bi-parental crosses were considered both as (1) individual populations as well as (2) condensed into four populations according to the parental cross. The second was performed because, while populations were obtained from separate F_1_ plants, the bi-parental cross was the same for Populations 2 and 3, and for Populations 4 and 5, so these were each combined into one dataset for an additional analysis. Previously mapped markers, as reported in Hulse-Kemp et al., were used to annotate markers for the expected chromosome group [11]. JoinMap Version 5.0 was used to construct linkage maps for each population [24]. Markers with a similarity score of 1.0 were removed. Grouping markers were run to a maximum logarithm of odds (LOD) score of 20. Groups of markers from the same chromosome (or groups containing some markers without a prior-mapped location or less than 3 markers disagreeing with the majority chromosome) that contained more than 10 markers were kept. Due to the small populations, the calculated centiMorgan (cM) distances were larger than expected, so the order of the genetic maps were compared to previous literature [11] using MapChart version 2.32 [25]. The comparison with previous literature was also utilized for identification of the cotton chromosome (using the 1–26 designation) that the linkage groups corresponded to. Additionally, markers were converted to .ped format and run with single marker association analysis with PLINK V1.9 [17,18], and statistical significance was evaluated at p-value level 0.05 with standard multiple testing correction.

### 4.5. Quantitative Trait Loci (QTL) Analysis

QTL mapping was completed using MapQTL Version 6 [26]. SNP data from the .loc file, the compiled map file generated from JoinMap, and the phenotypic data were used as input for the program. QTL were calculated using the Interval Mapping analysis. Different variations were analyzed for QTL for each of the populations and checked for similarity and strength of results due to the nature of the small populations and the fluidity of the phenotypic scale of disease. In addition to the default initial run with all raw data, (1) “Normal”—these variations included running the analysis using four different additional methods categorized as following: (2) “mapped only”—only the markers that were in linkage groups (MapQTL’s set of input files include the original JoinMap .loc file, which typically contains all of the markers that originally went into JoinMap; for this set, all of the markers that did not make it into the linkage groups were removed from this file); (3) “Data Regrouped [0,1] * [3,4]”—clustering similar phenotypes together such as 3 and 4 being denoted as the same phenotype; (4) “Data Regrouped [0] [1,4]”—changing the phenotypes to be either 0—no disease—or 4—any disease symptoms; and (5) “Real Loc”—exchanging the marker positions obtained in this study with those obtained by linkage mapping of larger-sized segregating positions, i.e., potentially more accurate positions, found in Hulse-Kemp (2015) [11]. Permutation tests were performed for 10,000 iterations to determine the LOD score for significant QTL.

### 4.6. Marker Assay Development and Validation

A small testing panel was created to test the markers associated with QTL in an assay to see if they are able to be validated to show segregation as expected for the different alleles across the populations (Table 4). The panel was designed to include several individuals from parental lines of the F_2_ populations (MD23ne, TX1145, TX1214, and Mac7-1238) including a few additional wild landraces (TX2425 and TX2452), lab created F_1_ samples (to ensure representatives in the heterozygous cluster, as no biological samples were available), and a set of 10 F_2_ individuals of the bi-parental crosses. Young leaf tissue samples were ground with a Pellet Pestle Motor from Fisher Scientific, and the DNA was extracted using the DNEasy Plant kit. The samples were quantified using SpectraMax QuickDrop Spectrophotometer (Molecular Devices) and Picogreen. All samples were standardized to 15 [ng/µL] for high DNA concentrations. The few samples that had lower concentrations were standardized to 10 [ng/µL]. F_1_ samples were created in the lab by mixing DNA from both parents. The testing panel was replicated in 96-well sections of each quadrant of three 384-well plates, with all unfilled wells acting as non-template controls with water (Supplementary Materials, Table S3).

A subset of the significant SNP markers that were identified as having a LOD greater than 3.0 and a few SNPs closely linked to those QTL were selected for validation. The SNPs were used to design KASP primers following the protocol detailed in Hulse-Kemp et al., 2015 with BatchPrimer3 [12]. KASP assays were run on the testing panel using the manufacturers’ suggested standard thermocycler protocol for 52 cycles. Plates were read starting at cycle 34 and every 3 cycles using the BMG LABtech PHERAstar Plus Plate Reader (Firmware version 1.43) and Software (version 5.30 R3). Data were visualized using the LGC Genomics KlusterCaller Software (version 3.4.1.39). Data from each of the SNPs were normalized according to their own subsets (the testing panel was small enough that one assay was run on each quadrant of the 384-well plate). SNPs from the assay were confirmed to be validated if there was reasonable differentiation between the clusters, with expected clustering of samples.

## 5. Conclusions

In conclusion, we have identified multiple sources of resistance to the cotton leaf curl virus (CLCuV) and performed the first genetic analyses on these materials. The identification of multiple QTL in different locations on the genome suggests there are multiple resistance genes present in geographically diverse cotton germplasm. Identification of these sources provides the basis for more durable resistance via the stacking of these resistance QTL. We have developed a R/Shiny App, iCottonQTL, which streamlines the process to QTL mapping from cotton genotyping arrays and subsequent deposit of data to the CottonGen database.

## Figures and Tables

**Table 1:** Populations utilized for genetic mapping.

Population	Cross ^a^	Size of F_2_ Population ^b^	Number of Polymorphic Markers ^c^
1	MD26ne × TX1214	36	7969
2	Mac7-1238 × TX1145	39	8600
3		48	
4	MD26ne × TX1145	35	8307
5		30	
6	MD26ne × Mac7-0238	66	5369

^a^ List of population crosses. Blue indicates the resistant parent and black indicates the susceptible parent. Underline indicates a photoperiod sensitive parent. ^b^ Number of samples in the cross. ^c^ Number of polymorphic markers for each cross.

**Table 2:** Quantitative Trait Loci identified for each of the genetic mapping populations.

Population ^a^	Cross	Chromosome	Number of Markers	% Explained	Effect Parent
1	MD26ne × TX1214	8	2	33–51.5	TX1214
2 & 3	Mac7-1238 × TX1145	9	2	30.4 & 17.2	TX1145
		10	7	17–18	
		15	1	18.5	
		21	1	18.4	
4	MD26ne × TX1145	0	0	0	N/A
4 & 5	MD26ne × TX1145	0	0	0	N/A
5	MD26ne × TX1145	16	6	53–55.4	TX1145
6	MD26ne × Mac7-0238	3	6	41.3–50.6	Mac7-0238
		5	3	39.3–40.5	
		16	2	39.3	

^a^ Population 1, MD26ne × TX1214, shown in the top row identified 2 markers from chromosome 8. Combined Populations 2 and 3, Mac7-1238 × TX1145, identified 11 markers from chromosomes 9, 10, 15, and 21. Population 5, MD26ne × TX1145, identified 6 markers from chromosome 16. Population 6, MD26ne × Mac7-0238, identified 11 markers from Chromosomes 3, 5, and 16. Additional detailed information is provided in Supplementary Materials, Table S2.

**Table 3:** KASP assay screening results for QTL-associated and flanking markers.

Marker	Cross	QTL ^a^	Linkage Group	Position (cM)	Good/Bad ^b^
i04503Gh	MD26ne × TX1214	Yes	36	0	GOOD
i60979Gt	MD26ne × TX1214	Yes	36	1.502	GOOD
i64854Gm	MD26ne × TX1214	No	36	32.553	GOOD
i01747Gh	MD26ne × TX1145	No	27	70.891	BAD
i01975Gh	MD26ne × TX1145	Yes	27	104.098	GOOD
i20534Gh	MD26ne × TX1145	Yes	27	105.866	GOOD
i38904Gh	MD26ne × TX1145	No	27	132.962	GOOD
i38317Gh	MD26ne × TX1145	No	27	132.962	BAD
i35622Gh	MD26ne × TX1145	Yes	27	134.712	GOOD *
i41454Gh	MD26ne × TX1145	Yes	27	134.712	BAD
i01767Gh	MD26ne × TX1145	No	27	147.669	GOOD

^a^ QTL indicated as “No” represents flanking markers. ^b^ QTL marked as Good/Bad indicate whether the KASP markers separated into clusters sufficiently. * Might be best used as a dominant marker.

**Table 4:** Testing panel for validation of KASP markers associated with cotton leaf curl virus (CLCuV) Quantitative Trait Loci.

Sample Class	Entries ^b^	Biological Reps/Sample (#)	Technical Reps/Sample (#)
Parents	MD26ne, TX1145, TX1214, TX2425, TX2452, Mac7-1238	3,4,4,2,2,3	2,2,2,2,2,2
^a^ Synthetic F_1_s	MD26ne × TX1145, Mac7-1238 × MD26ne, MD26ne × TX1214, Mac7-1238 × TX1145	3,3,3,3	0,0,0,0
F_2_s	MD26ne × TX1145, MD26ne × TX1214, Mac7-1238 × TX1145	10,10,10	0,0,0

^a^ Synthetic F_1_ samples were generated by mixing equal amounts of parent samples as indicated. ^b^ Plate layout is shown in Supplementary Materials, Table S3.

## Data Availability

The data presented in this study are openly available in Github (https://github.com/USDA-ARS-GBRU/Cotton_CottonLeafCurlVirus_QTLmapping (accessed on 21 February 2023)) and on CottonGen (https://cottongen.org (accessed on 21 February 2023)).

## Supplementary Materials

The following supporting information can be downloaded at: https://www.mdpi.com/article/10.3390/plants12051153/s1, Figure S1: Distribution of Phenotypes for Population and Combined Populations; Figure S2: Linkage mapping marker synteny; Figure S3: Visualization of the KASP Markers that had reasonable clustering; Figure S4: Principal Component Analysis plots of the individuals from combined populations 2–5; Table S1: Details of Linkage Groups for each population; Table S2: Significant Markers across all populations; Table S3: Plate layout for the testing plate for the KASP markers.
