# Supplementary material for: Detecting Cotton Leaf Curl Virus Resistance Quantitative Trait Loci in Gossypium hirsutum and iCottonQTL a New R/Shiny App to Streamline Genetic Mapping
Source: Plants (Basel). 2023 Mar 3;12(5):1153. doi: 10.3390/plants12051153 (PMC10005503; doi:10.3390/plants12051153)
Supplement: Supplementary file 1 [file plants-12-01153-s001.zip › plants-2184499-proof done-figures.pdf]

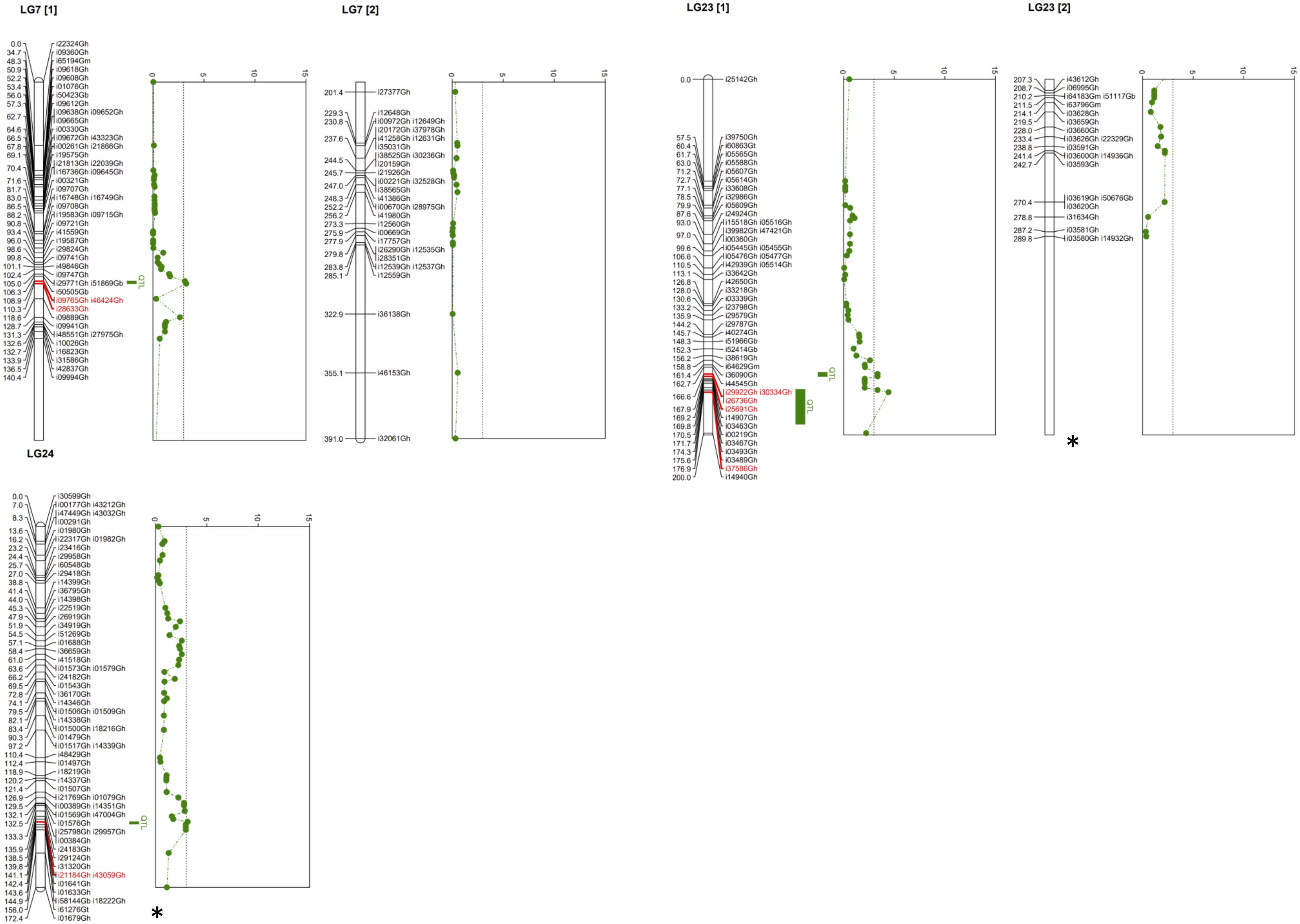
LG7 [1]
LG7 [2]
LG23 [1]
LG23 [2]
LG24
QTL
0
5
10
15
*

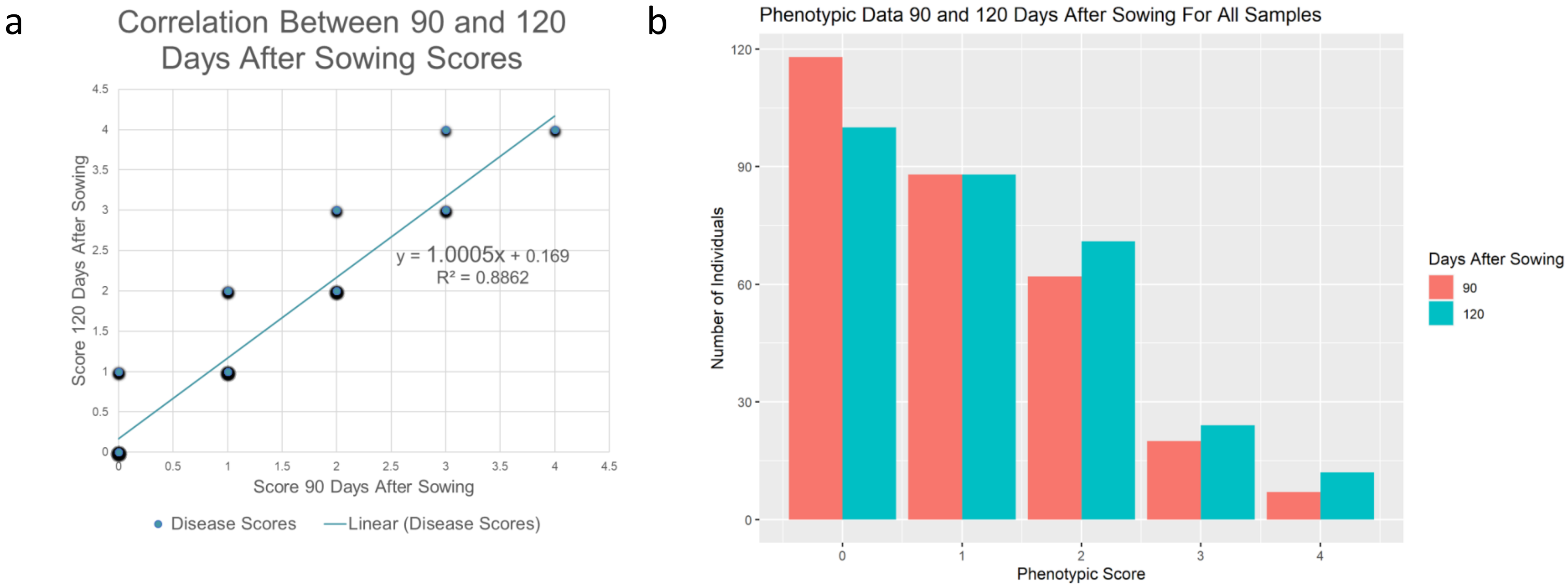
a
Correlation Between 90 and 120 Days After Sowing Scores
y = 1.0005x + 0.169
R² = 0.8862
Score 120 Days After Sowing
Score 90 Days After Sowing
0
0.5
1
1.5
2
2.5
3
3.5
4
4.5
Disease Scores
Linear (Disease Scores)
b
Phenotypic Data 90 and 120 Days After Sowing For All Samples
Number of Individuals
0
30
60
90
120
Phenotypic Score
0
1
2
3
4
Days After Sowing
90
120

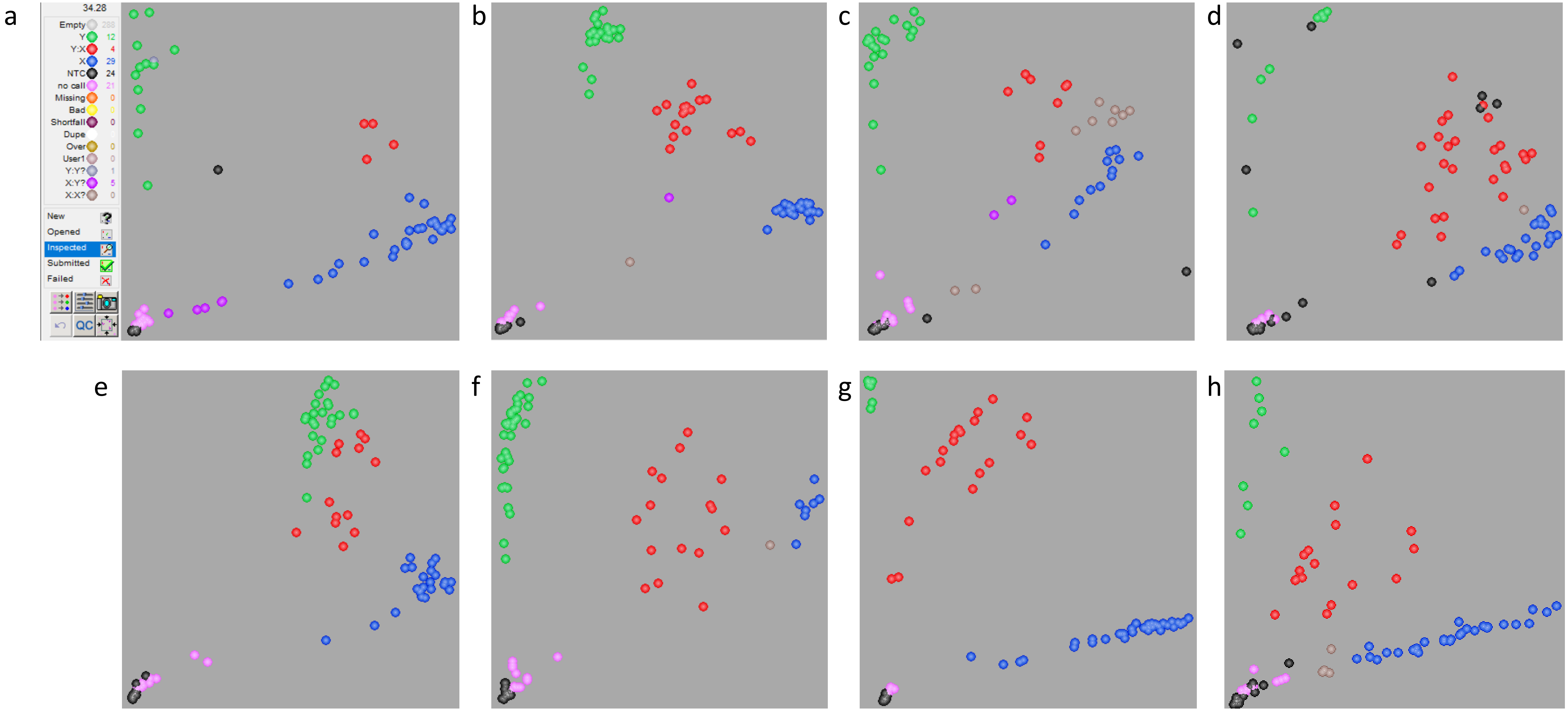
a
b
c
d
e
f
g
h
34.28
Empty 288
Y 12
Y:X 4
X 29
NTC 24
no call 21
Missing 0
Bad 0
Shortfall 0
Dupe
Over 0
User1 0
Y:Y? 1
X:Y? 5
X:X? 0
New
Opened
Inspected
Submitted
Failed
QC

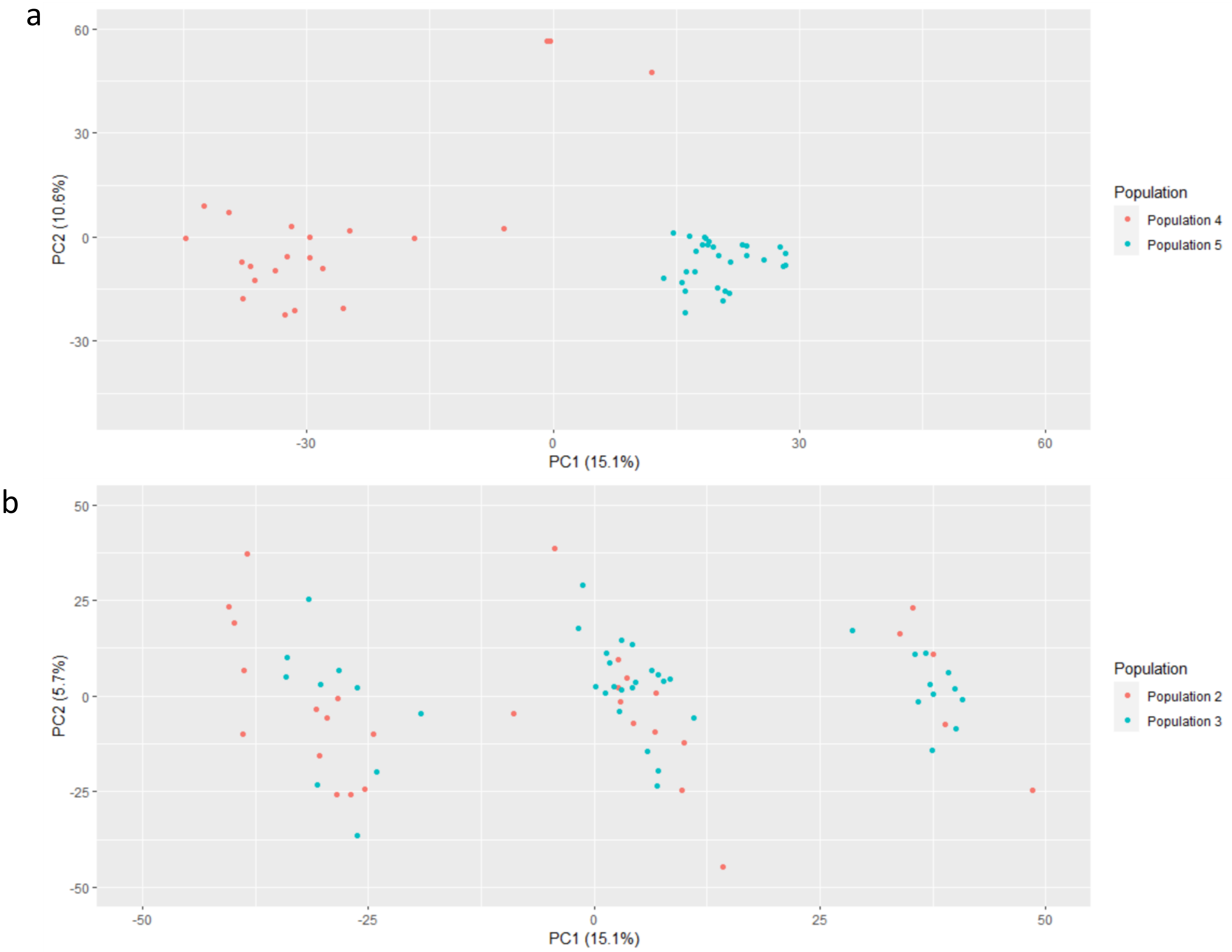
a
PC2 (10.6%)
60
30
0
-30
-30
0
30
60
PC1 (15.1%)
Population
Population 4
Population 5
b
PC2 (5.7%)
50
25
0
-25
-50
-50
-25
0
25
50
PC1 (15.1%)
Population
Population 2
Population 3

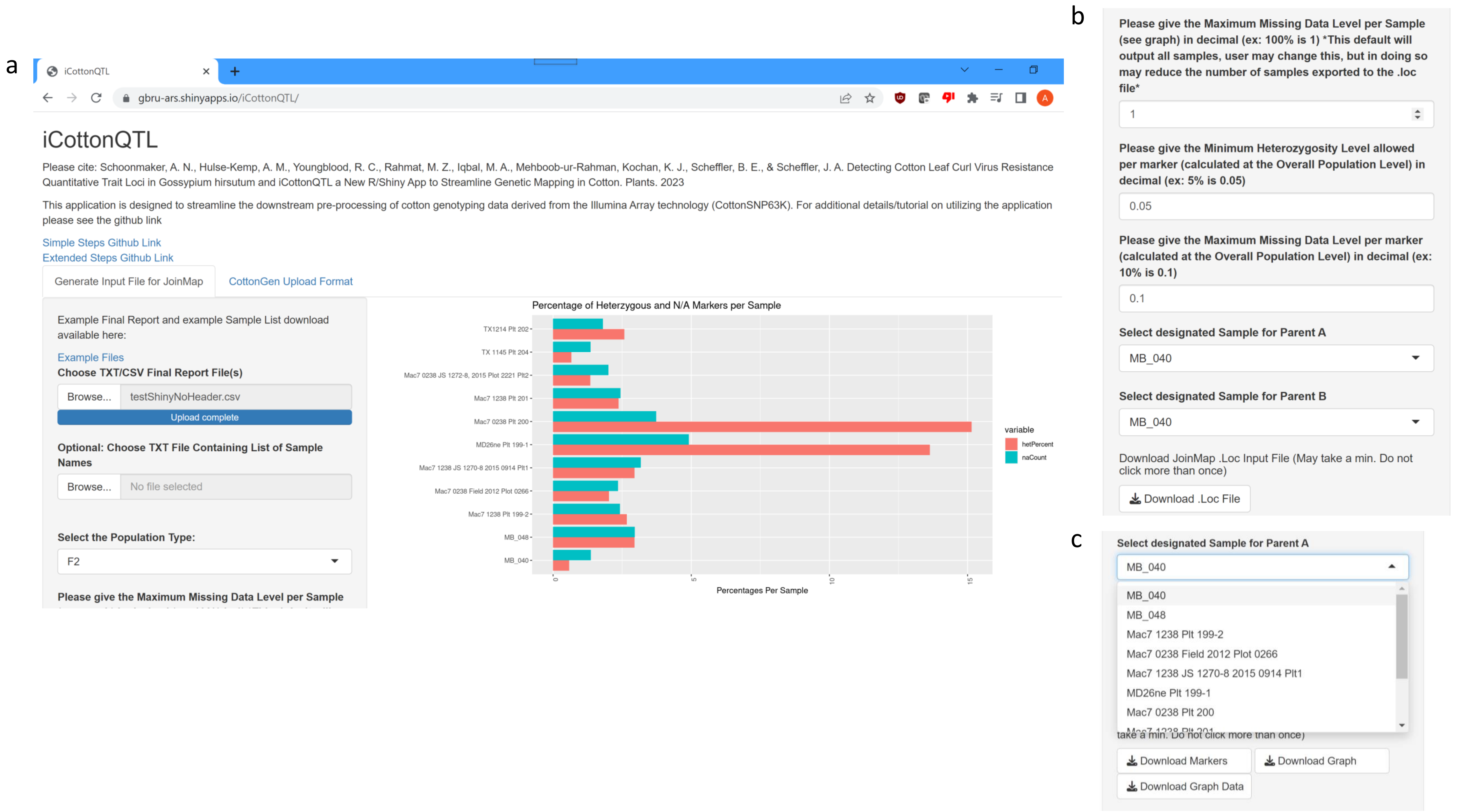
a
iCottonQTL
gbru-ars.shinyapps.io/iCottonQTL/
iCottonQTL
Please cite: Schoonmaker, A. N., Hulse-Kemp, A. M., Youngblood, R. C., Rahmat, M. Z., Iqbal, M. A., Mehboob-ur-Rahman, Kochan, K. J., Scheffler, B. E., & Scheffler, J. A. Detecting Cotton Leaf Curl Virus Resistance Quantitative Trait Loci in Gossypium hirsutum and iCottonQTL a New R/Shiny App to Streamline Genetic Mapping in Cotton. Plants. 2023
This application is designed to streamline the downstream pre-processing of cotton genotyping data derived from the Illumina Array technology (CottonSNP63K). For additional details/tutorial on utilizing the application please see the github link
Simple Steps Github Link
Extended Steps Github Link
Generate Input File for JoinMap
CottonGen Upload Format
Example Final Report and example Sample List download available here:
Example Files
Choose TXT/CSV Final Report File(s)
Browse...
testShinyNoHeader.csv
Upload complete
Optional: Choose TXT File Containing List of Sample Names
Browse...
No file selected
Select the Population Type:
F2
Please give the Maximum Missing Data Level per Sample
Percentage of Heterzygous and N/A Markers per Sample
TX1214 Plt 202
TX 1145 Plt 204
Mac7 0238 JS 1272-8, 2015 Plot 2221 Plt2
Mac7 1238 Plt 201
Mac7 0238 Plt 200
MD26ne Plt 199-1
Mac7 1238 JS 1270-8 2015 0914 Plt1
Mac7 0238 Field 2012 Plot 0266
Mac7 1238 Plt 199-2
MB_048
MB_040
0
5
10
15
Percentages Per Sample
variable
hetPercent
naCount
b
Please give the Maximum Missing Data Level per Sample (see graph) in decimal (ex: 100% is 1) *This default will output all samples, user may change this, but in doing so may reduce the number of samples exported to the .loc file*
1
Please give the Minimum Heterozygosity Level allowed per marker (calculated at the Overall Population Level) in decimal (ex: 5% is 0.05)
0.05
Please give the Maximum Missing Data Level per marker (calculated at the Overall Population Level) in decimal (ex: 10% is 0.1)
0.1
Select designated Sample for Parent A
MB_040
Select designated Sample for Parent B
MB_040
Download JoinMap .Loc Input File (May take a min. Do not click more than once)
Download .Loc File
c
Select designated Sample for Parent A
MB_040
MB_040
MB_048
Mac7 1238 Plt 199-2
Mac7 0238 Field 2012 Plot 0266
Mac7 1238 JS 1270-8 2015 0914 Plt1
MD26ne Plt 199-1
Mac7 0238 Plt 200
take a min. Do not click more than once)
Download Markers
Download Graph
Download Graph Data

d

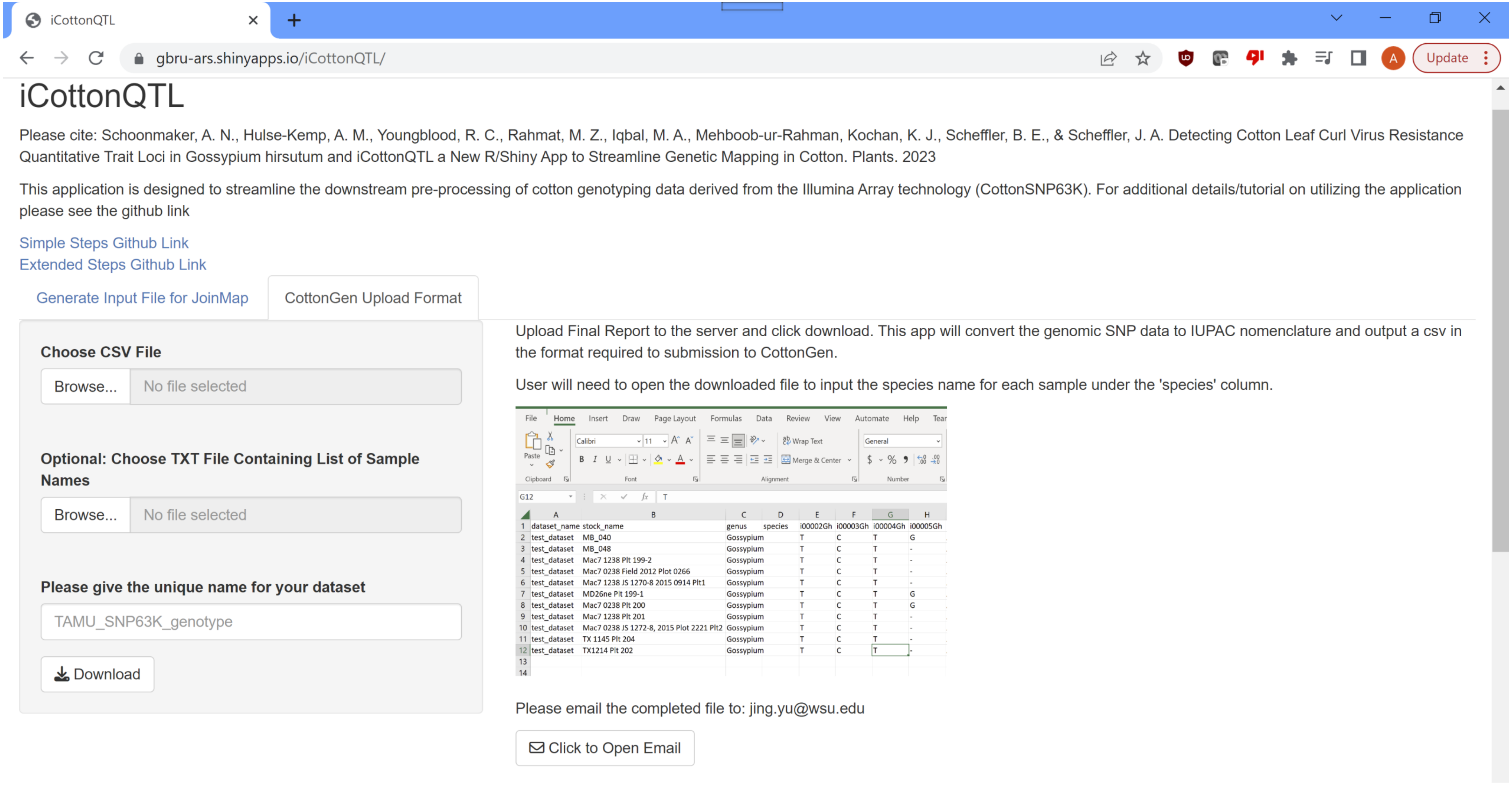
iCottonQTL
gbru-ars.shinyapps.io/iCottonQTL/
Update
iCottonQTL
Please cite: Schoonmaker, A. N., Hulse-Kemp, A. M., Youngblood, R. C., Rahmat, M. Z., Iqbal, M. A., Mehboob-ur-Rahman, Kochan, K. J., Scheffler, B. E., & Scheffler, J. A. Detecting Cotton Leaf Curl Virus Resistance Quantitative Trait Loci in Gossypium hirsutum and iCottonQTL a New R/Shiny App to Streamline Genetic Mapping in Cotton. Plants. 2023
This application is designed to streamline the downstream pre-processing of cotton genotyping data derived from the Illumina Array technology (CottonSNP63K). For additional details/tutorial on utilizing the application please see the github link
Simple Steps Github Link
Extended Steps Github Link
Generate Input File for JoinMap
CottonGen Upload Format
Choose CSV File
Browse...
No file selected
Optional: Choose TXT File Containing List of Sample Names
Browse...
No file selected
Please give the unique name for your dataset
TAMU_SNP63K_genotype
Download
Upload Final Report to the server and click download. This app will convert the genomic SNP data to IUPAC nomenclature and output a csv in the format required to submission to CottonGen.
User will need to open the downloaded file to input the species name for each sample under the 'species' column.
dataset_name stock_name genus species i00002Gh i00003Gh i00004Gh i00005Gh
test_dataset MB_040 Gossypium T C T G
test_dataset MB_048 Gossypium T C T -
test_dataset Mac7 1238 Plt 199-2 Gossypium T C T -
test_dataset Mac7 0238 Field 2012 Plot 0266 Gossypium T C T -
test_dataset Mac7 1238 JS 1270-8 2015 0914 Plt1 Gossypium T C T -
test_dataset MD26ne Plt 199-1 Gossypium T C T G
test_dataset Mac7 0238 Plt 200 Gossypium T C T G
test_dataset Mac7 1238 Plt 201 Gossypium T C T -
test_dataset Mac7 0238 JS 1272-8, 2015 Plot 2221 Plt2 Gossypium T C T -
test_dataset TX 1145 Plt 204 Gossypium T C T -
test_dataset TX1214 Plt 202 Gossypium T C T -
Please email the completed file to: jing.yu@wsu.edu
Click to Open Email
