# Supplementary material for: Detecting Cotton Leaf Curl Virus Resistance Quantitative Trait Loci in Gossypium hirsutum and iCottonQTL a New R/Shiny App to Streamline Genetic Mapping
Source: Plants (Basel). 2023 Mar 3;12(5):1153. doi: 10.3390/plants12051153 (PMC10005503; doi:10.3390/plants12051153)
Supplement: Supplementary file 1 [file plants-12-01153-s001.zip › plants-2184499-Supplementary.pdf]

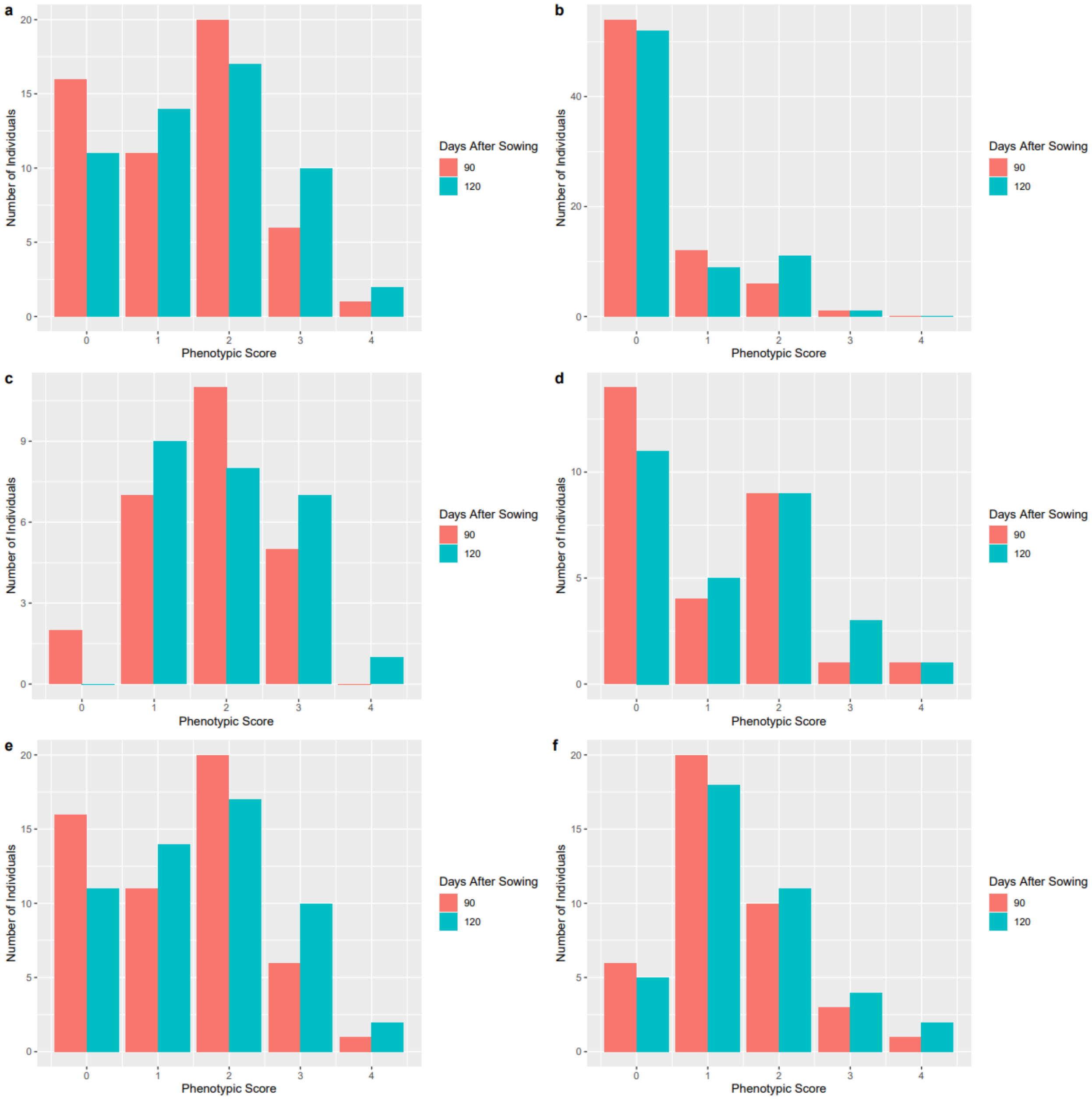

Figure S1: Distribution of Phenotypes for Population and Combined Populations. The number of individuals for each phenotypic score at 90 Days after Sowing (DAS) shown in blue and 120 DAS shown in gray in each graph. Phenotypic distributions shown for (**a**) Population 1, (**b**) Population 2 and 3, (**c**) Population 4, (**d**) Population 5, (**e**) Population 4 and 5, and (**f**) Population 6.

Table S1: Details of Linkage Groups for each population. Number of linkage groups, total markers, and total length of linkage groups are listed by chromosome for each mapping population.

Population	Chromosome	# of Groups	Total # of Loci	Total Contig Length
1	1	1	275	826.9
1	2	3	94	289.6
1	3	3	123	425
1	4	1	148	831.5
1	5	4	257	969.6
1	6	3	209	703.8
1	7	1	41	164.6
1	8	3	227	612.4
1	9	3	190	810.1
1	10	3	209	810.3
1	11	4	270	1169.9
1	12	1	217	1114.4
1	13	2	234	691.5
1	14	5	188	725.8
1	15	2	163	669.3
1	16	3	256	966.6
1	17	4	89	386
1	18	2	222	811.5
1	19	5	335	1385.2
1	20	2	282	1057.4
1	21	4	210	917.3
1	22	3	126	579.1
1	23	1	218	874.6
1	24	4	253	810.6
1	25	2	209	716.2
1	26	1	295	1069.94
2&3	1	1	57	535
2&3	2	1	52	10272.9
2&3	3	1	303	1004.6
2&3	4	1	164	737.6
2&3	5	1	366	1309.2
2&3	6	1	157	608.9
2&3	7	1	294	942
2&3	8	1	477	1038
2&3	9	1	129	645
2&3	10	1	307	918.6
2&3	11	1	307	1066
2&3	12	1	220	994

2&3	13	2	57	191.7
2&3	14	1	345	978.7
2&3	15	1	219	777.8
2&3	16	4	345	858.4
2&3	17	1	214	585
2&3	18	2	246	745.6
2&3	19	2	212	805
2&3	20	0		
2&3	21	2	198	714.7
2&3	22	1	94	483.5
2&3	23	2	123	571.5
2&3	24	1	375	1071
2&3	25	1	219	744.9
2&3	26	1	217	1100.9
4	1	2	140	603.9
4	2	2	64	156
4	3	3	76	353.5
4	4	2	74	393.5
4	5	7	146	734.6
4	6	6	87	237.9
4	7	5	102	356.2
4	8	5	123	356.4
4	9	5	115	368
4	10	3	107	447
4	11	6	100	286.48
4	12	4	99	379.9
4	13	2	58	186.6
4	14	2	108	10318.7
4	15	4	81	383.9
4	16	6	106	253.5
4	17	3	56	129.4
4	18	3	81	284.5
4	19	4	95	355
4	20	4	121	374.8
4	21	6	100	458.6
4	22	1	31	83.6
4	23	5	114	324.7
4	24	1	123	457.59
4	25	3	94	411.4
4	26	3	106	373.6
5	1	1	147	274.2
5	2	1	43	188.4
5	3	1	113	10302.2
5	4	2	50	142.7
5	5	4	111	10450.3

5	6	3	71	216.3
5	7	2	58	217
5	8	2	91	10244.1
5	9	4	95	313.6
5	10	2	94	358.8
5	11	7	129	10195.5
5	12	4	90	10277
5	13	1	131	415.9
5	14	1	106	372.2
5	15	2	70	287
5	16	2	65	322.8
5	17	3	62	190.4
5	18	2	88	377.3
5	19	2	129	450.1
5	20	3	125	259.6
5	21	2	87	291.2
5	22	1	83	316.7
5	23	1	71	304.4
5	24	1	127	397.8
5	25	1	82	247.9
5	26	1	106	492.1
4&5	1	2	270	605
4&5	2	3	102	242
4&5	3	1	168	479
4&5	4	1	114	461
4&5	5	3	180	868
4&5	6	4	161	561
4&5	7	3	145	429
4&5	8	3	201	410
4&5	9	2	172	618
4&5	10	2	172	694.3
4&5	11	2	234	527
4&5	12	1	136	662
4&5	13	3	238	446.8
4&5	14	3	192	524
4&5	15	2	129	485
4&5	16	3	208	636.2
4&5	17	2	113	400
4&5	18	2	164	495
4&5	19	4	245	716.5
4&5	20	2	231	678
4&5	21	1	173	940
4&5	22	2	129	506.5
4&5	23	2	143	482
4&5	24	2	242	570

4&5	25	1	152	462
4&5	26	1	216	730
6	1	2	156	612.4
6	2	1	81	251.6
6	3	1	70	402.3
6	4	2	21	90.3
6	5	2	106	490.4
6	6	2	90	366.4
6	7	2	83	358.9
6	8	1	297	839
6	9	3	131	445.1
6	10	1	46	216.6
6	11	3	116	525.6
6	12	2	66	382.6
6	13	2	152	578.1
6	14	2	96	311.2
6	15	2	56	349.3
6	16	2	78	10295
6	17	1	15	100
6	18	1	33	173
6	19	2	119	552.2
6	20	1	21	78
6	21	1	72	381.8
6	22	1	106	477.2
6	23	1	118	399.2
6	24	1	42	236
6	25	1	32	282
6	26	2	42	319.2

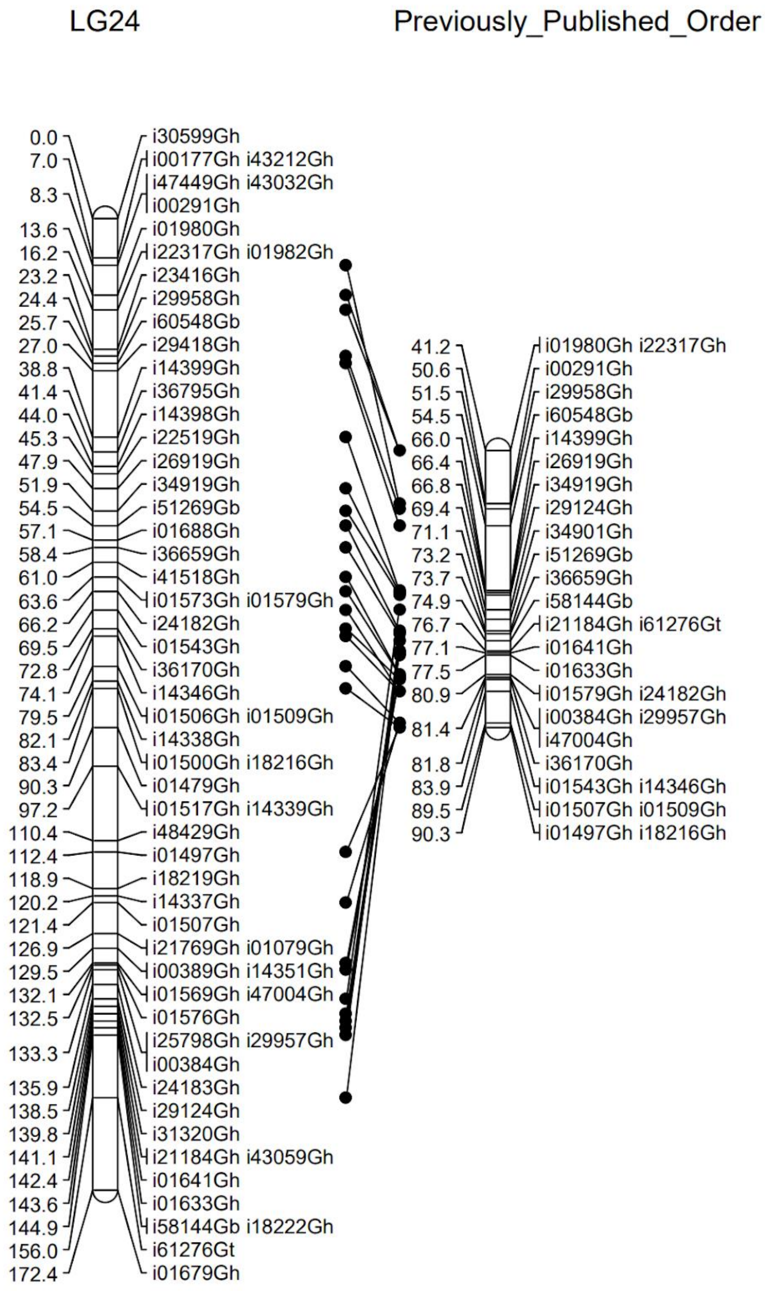

Figure S2: Linkage mapping marker synteny. MapChart comparison of linkage group order from this study (left) with previously published linkage group order (right) [11]. Distances are listed in centiMorgans. Example shown for LG24 from Population 6.

Table S2: Significant Markers across all populations. Test Run ID indicates the method of grouping the phenotypic score and JoinMap results. Besides the typical method with all raw data 1) "Normal", the rest of the test run IDs are running the analysis using four different additional methods categorized as following: 2) "mapped only" - only the markers that were in linkage groups (as MapQTL's input files require the original JoinMap .loc file which typically contains all of the markers that originally went into JoinMap, for this set all of the markers that did not make it into the linkage groups were also removed from this file); 3) "Data Regrouped [0,1] * [3,4]" - clustering similar phenotypes together such as 3 and 4 being denoted as the same phenotype; 4) "Data Regrouped [0] [1,4]" - changing the phenotypes to be either 0 – no disease – or 4 – having the disease; and 5) "Real Loc" - exchanging the marker positions obtained in this study with those obtained by linkage mapping of larger-sized segregating positions, ie. potentially more accurate positions, found in Hulse-Kemp et al. (2015). The marker group and marker group position refer to the group number from the JoinMap results. Genome Chromosome refers back to the appropriate chromosome the group maps back to.

Test Run ID	Population	Marker	Marker Group #	Marker Group Position	LOD	Genome Chromosome	Variance	Percent Explained	Allele Parent
Normal	1	i60979Gt	36	1.502	3.23	8	0.82684	33.8	TX1214
Data Regrouped [0,1] * [3,4]	1	i04503Gh	36	0	3.45	8	1.36385	51.5	TX1214
Normal	2 and 3	i28523Gh	6	805.34	3.09	10	0.520394	17.7	TX1145
Normal	2 and 3	i18794Gh	10	76.495	3	24	0.523349	17.2	TX1145
Data Regrouped [0,1] * [3,4]	2 and 3	i19232Gh	27	450.205	4.87	9	0.176788	30.4	TX1145
Real Loc	2 and 3	i54072Gb	6	117.2	3.09	10	0.520394	17.7	TX1145
Real Loc	2 and 3	i61540Gt	6	117.6	3.02	10	0.522684	17.3	TX1145
Real Loc	2 and 3	i63736Gm	6	117.6	3.02	10	0.522684	17.3	TX1145
Data Regrouped [0] [1,4]	2 and 3	i11809Gh	6	318.488	3.03	10	2.70861	17.4	TX1145
Data Regrouped [0] [1,4]	2 and 3	i40202Gh	6	201.424	3.28	10	2.66535	18.7	TX1145
Data Regrouped [0] [1,4]	2 and 3	i29192Gh	6	795.582	3.17	10	2.68485	18.1	TX1145
Data Regrouped [0] [1,4]	2 and 3	i60707Gt	21	486.09	3.25	15	2.67096	18.5	TX1145

Data Regrouped [0] [1,4]	2 and 3	i19282Gh	24	501.557	3.22	21	2.67589	18.4	TX1145
Data Regrouped [0,1] * [3,4]	5	i37027Gh	27	104.098	3.5	16	1.14286	55.4	TX1145
Data Regrouped [0,1] * [3,4]	5	i01975Gh	27	104.098	3.5	16	1.14286	55.4	TX1145
Data Regrouped [0,1] * [3,4]	5	i35622Gh	27	134.712	3.5	16	1.14286	55.4	TX1145
Data Regrouped [0,1] * [3,4]	5	i00262Gh	27	134.712	3.5	16	1.14286	55.4	TX1145
Data Regrouped [0,1] * [3,4]	5	i41454Gh	27	134.712	3.5	16	1.14286	55.4	TX1145
Data Regrouped [0,1] * [3,4]	5	i20534Gh	27	105.866	3.29	16	1.2	53.1	TX1145
Data Regrouped [0,1] * [3,4]	6	i37586Gh	23	176.87	4.44	3	1.29746	50.6	Mac7-0238
Data Regrouped [0,1] * [3,4]	6	i29922Gh	23	166.646	3.36	3	1.54023	41.3	Mac7-0238
Data Regrouped [0,1] * [3,4]	6	i30334Gh	23	166.646	3.36	3	1.54023	41.3	Mac7-0238
Data Regrouped [0,1] * [3,4]	6	i26736Gh	23	166.646	3.36	3	1.54023	41.3	Mac7-0238
Data Regrouped [0,1] * [3,4]	6	i25691Gh	23	167.919	3.36	3	1.54023	41.3	Mac7-0238
Data Regrouped [0,1] * [3,4]	6	i03489Gh	23	175.596	3.36	3	1.54023	41.3	Mac7-0238
Data Regrouped [0,1] * [3,4]	6	i28633Gh	7	110.34	3.27	5	1.56305	40.5	Mac7-0238
Data Regrouped [0,1] * [3,4]	6	i09765Gh	7	108.861	3.14	5	1.59387	39.3	Mac7-0238

Data Regrouped [0,1] * [3,4]	6	i46424Gh	7	108.861	3.14	5	1.59387	39.3	Mac7-0238
Data Regrouped [0,1] * [3,4]	6	i21184Gh	24	141.096	3.14	16	1.59387	39.3	Mac7-0238
Data Regrouped [0,1] * [3,4]	6	i43059Gh	24	141.096	3.14	16	1.59387	39.3	Mac7-0238

Table S3: Plate layout for the testing plate for the KASP markers. Rep indicates technical replicate of the sample originally found, such as rep A1 indicates technical replicate of cell A1 sample. NTC indicates non-template control.

	**1**	**2**	**3**	**4**	**5**	**6**	**7**	**8**	**9**	**10**	**11**	**12**
**A**	MD26ne	rep H1 - TX 1145	TX1214	TX2425	MD26ne x TX1145 F1	Mac7 - 1238 x TX1145 F1	MD26ne x TX1145 F2	MD26ne x TX1214 F2	Mac7 - 1238 x TX1145 F2	NTC	NTC	NTC
**B**	rep A1 - MD26ne	TX 1145	Mac7 - 1238	rep A4 - TX 2425	Mac7-1238 x MD26ne F1	Mac7 - 1238 x TX1145 F1	MD26ne x TX1145 F2	MD26ne x TX1214 F2	Mac7 - 1238 x TX1145 F2	NTC	NTC	NTC
**C**	MD26ne	TX 1145	rep B3 - Mac7 -1238	TX 2452	Mac7-1238 x MD26ne F1	MD26ne x TX1145 F2	MD26ne x TX1145 F2	MD26ne x TX1214 F2	Mac7 - 1238 x TX1145 F2	NTC	NTC	NTC
**D**	rep C1 - MD26ne	TX1214	Mac7 - 1238	rep C4 - TX 2452	Mac7-1238 x MD26ne F1	MD26ne x TX1145 F2	MD26ne x TX1145 F2	MD26ne x TX1214 F2	Mac7 - 1238 x TX1145 F2	NTC	NTC	NTC
**E**	MD26ne	rep D2 - TX 1214	rep D3 - Mac7 -1238	TX 2452	MD26ne x TX1214 F1	MD26ne x TX1145 F2	MD26ne x TX1214 F2	MD26ne x TX1214 F2	Mac7 - 1238 x TX1145 F2	NTC	NTC	NTC
**F**	TX 1145	TX1214	Mac7 - 1238	rep E4 - TX 2452	MD26ne x TX1214 F1	MD26ne x TX1145 F2	MD26ne x TX1214 F2	MD26ne x TX1214 F2	Mac7 - 1238 x TX1145 F2	NTC	NTC	NTC
**G**	rep F1 - TX 1145	rep F2 - TX 1214	TX2425	MD26ne x TX1145 F1	MD26ne x TX1214 F1	MD26ne x TX1145 F2	MD26ne x TX1214 F2	Mac7 - 1238 x TX1145 F2	Mac7 - 1238 x TX1145 F2	NTC	NTC	NTC
**H**	TX 1145	TX1214	rep G3 - TX 2425	MD26ne x TX1145 F1	Mac7 - 1238 x TX1145 F1	MD26ne x TX1145 F2	MD26ne x TX1214 F2	Mac7 - 1238 x TX1145 F2	Mac7 - 1238 x TX1145 F2	NTC	NTC	NTC

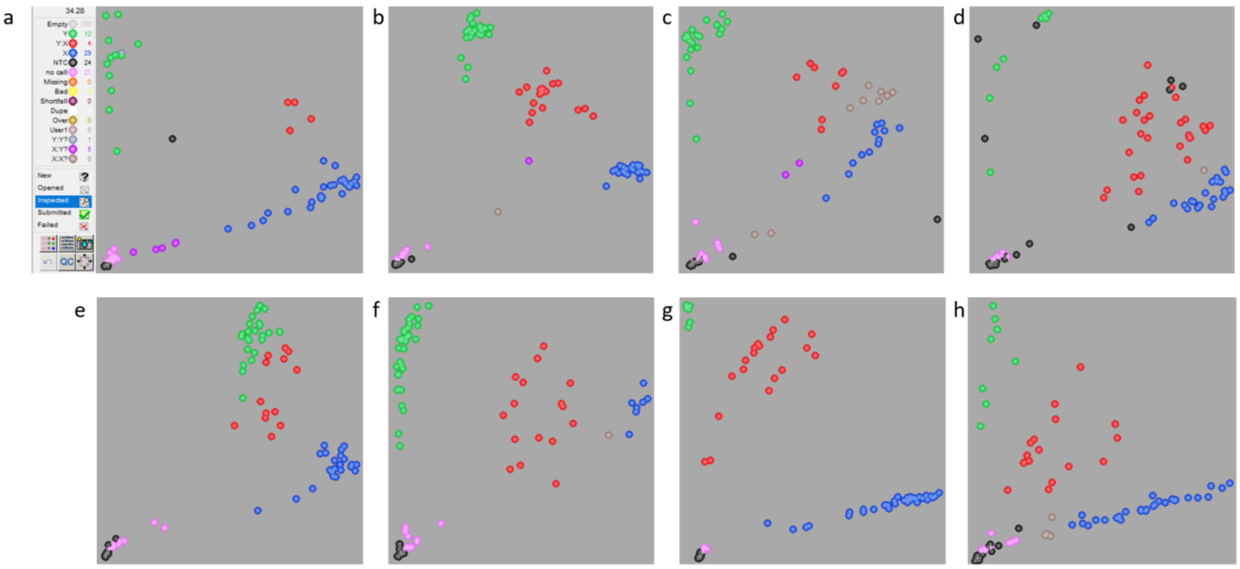

Figure S3: Visualization of the KASP Markers that had reasonable clustering. Markers in order are (**a**) i38904Gh, (**b**) i20534Gh, (**c**) i01975Gh, (**d**) i01767Gh, (**e**) i35622Gh, (**f**) i64854Gm, (**g**) i60979Gt, and (**h**) i04503Gh. Legend for all shown to the left of the (**a**).

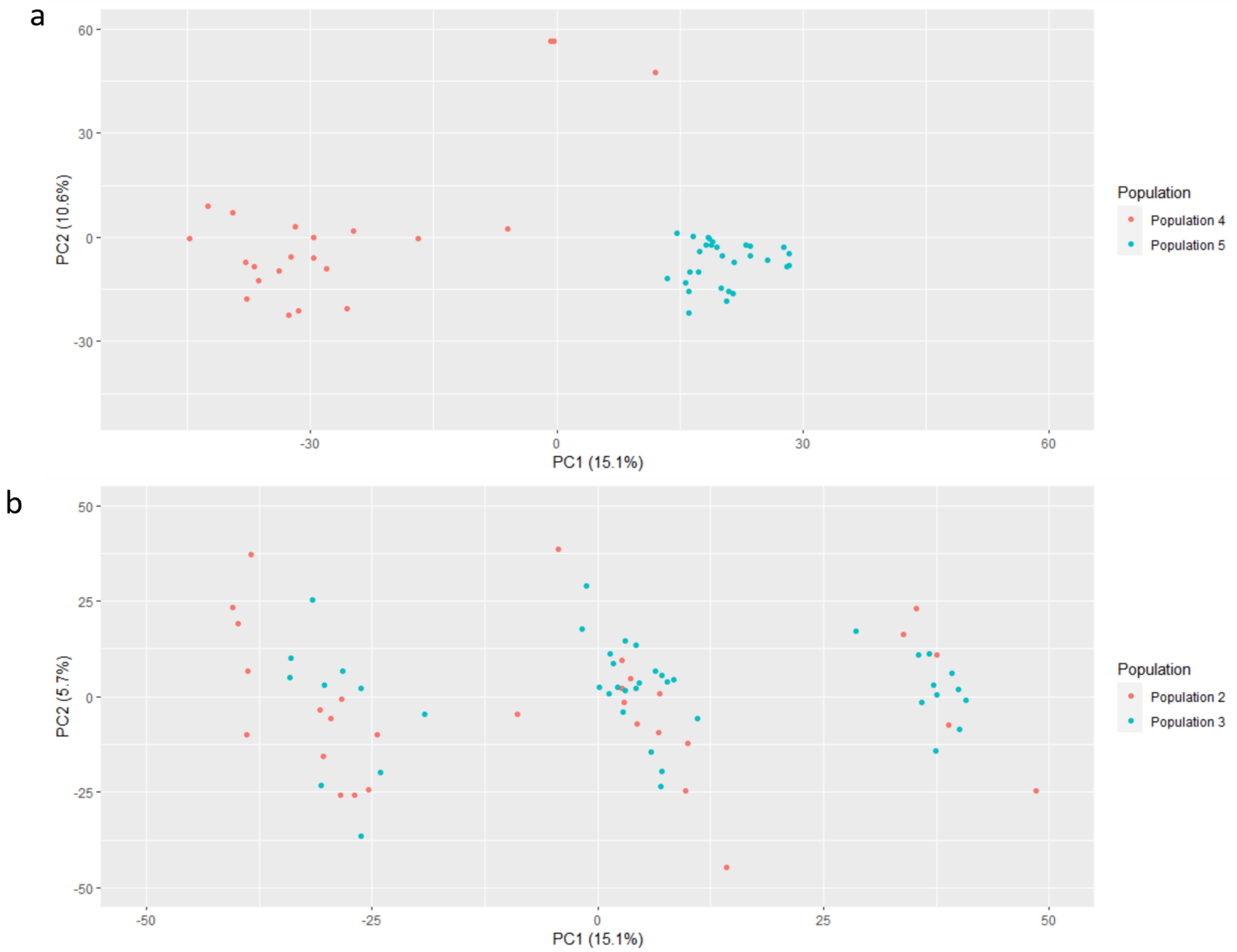

Figure S4: Principal Component Analysis plots of the individuals from combined populations 2-5. For each population, markers were filtered according to their status of polymorphism between the two parents' homozygous markers. The F2 genetic data was assigned into A, B, and H format for each SNP according to whether the SNP was homozygous matching parent A, parent B, or was heterozygous. ABH format SNPs were assigned numerical values of 0, 2, and 1 respectively. Missing values were assigned the average of the known values for its respective SNP. (**a**) PCA of the combined Populations 2 and 3. (**b**) PCA of the combined Populations 4 and 5.
